# One-Step Fabrication of Novel Polyethersulfone-Based Composite Electrospun Nanofiber Membranes for Food Industry Wastewater Treatment

**DOI:** 10.3390/membranes12040413

**Published:** 2022-04-11

**Authors:** Md. Nahid Pervez, Md Eman Talukder, Monira Rahman Mishu, Antonio Buonerba, Pasquale Del Gaudio, George K Stylios, Shadi W. Hasan, Yaping Zhao, Yingjie Cai, Alberto Figoli, Tiziano Zarra, Vincenzo Belgiorno, Hongchen Song, Vincenzo Naddeo

**Affiliations:** 1 Sanitary Environmental Engineering Division (SEED), Department of Civil Engineering, University of Salerno, 84084 Fisciano, Italy; mpervez@unisa.it (M.N.P.); abuonerba@unisa.it (A.B.); tzarra@unisa.it (T.Z.); v.belgiorno@unisa.it (V.B.); 2 Shenzhen Institute of Advanced Technology, Chinese Academy of Sciences, Shenzhen 518055, China; 2654410096@mails.ucas.ac.cn; 3 Institute on Membrane Technology (CNR-ITM), Via P. Bucci 17/c, 87036 Rende, Italy; a.figoli@itm.cnr.it; 4 Faculty of Nutrition and Food Science, Patuakhali Science and Technology University, Patuakhali 8602, Bangladesh; monirarmishu@gmail.com; 5 Department of Pharmacy, University of Salerno, Via Giovanni Paolo II 132, 84084 Fisciano, Italy; pdelgaudio@unisa.it; 6 Research Institute for Flexible Materials, School of Textiles and Design, Heriot-Watt University, Galashiels TD1 3HF, UK; g.stylios@hw.ac.uk; 7 Department of Chemical Engineering, Khalifa University of Science and Technology, Abu Dhabi P.O. Box 127788, United Arab Emirates; shadi.hasan@ku.ac.ae; 8 Institute of Eco-Chongming, School of Ecological and Environmental Sciences, East China Normal University, Shanghai 200241, China; ypzhao@des.ecnu.edu.cn; 9 Hubei Provincial Engineering Laboratory for Clean Production and High Value Utilization of Bio-Based Textile Materials, Wuhan Textile University, Wuhan 430200, China; yingjiecai@wtu.edu.cn

**Keywords:** polyethersulfone, hydroxypropyl cellulose, electrospun nanofiber membrane, food industry wastewater, adsorption

## Abstract

Using an environmentally friendly approach for eliminating methylene blue from an aqueous solution, the authors developed a unique electrospun nanofiber membrane made of a combination of polyethersulfone and hydroxypropyl cellulose (PES/HPC). SEM results confirmed the formation of a uniformly sized nanofiber membrane with an ultrathin diameter of 168.5 nm (for PES/HPC) and 261.5 nm (for pristine PES), which can be correlated by observing the absorption peaks in FTIR spectra and their amorphous/crystalline phases in the XRD pattern. Additionally, TGA analysis indicated that the addition of HPC plays a role in modulating their thermal stability. Moreover, the blended nanofiber membrane exhibited better mechanical strength and good hydrophilicity (measured by the contact angle). The highest adsorption capacity was achieved at a neutral pH under room temperature (259.74 mg/g), and the pseudo-second-order model was found to be accurate. In accordance with the Langmuir fitted model and MB adsorption data, it was revealed that the adsorption process occurred in a monolayer form on the membrane surface. The adsorption capacity of the MB was affected by the presence of various concentrations of NaCl (0.1–0.5 M). The satisfactory reusability of the PES/HPC nanofiber membrane was revealed for up to five cycles. According to the mechanism given for the adsorption process, the electrostatic attraction was shown to be the most dominant in increasing the adsorption capacity. Based on these findings, it can be concluded that this unique membrane may be used for wastewater treatment operations with high efficiency and performance.

## 1. Introduction

The pollution of the world’s water supplies has been noted as a major issue. Contaminants in wastewater may include a wide range of substances, such as microbes, colloids, proteins, heavy metals, and dyestuffs. Among them, dyestuff-contaminated industrial wastewater has significantly negatively impacted water and land quality, human health, and the ecosystem. It is the most challenging compound to remove from the industrial effluent streams because of having a stable and complex structure [1,2,3,4,5]. Notably, the food industry uses a variety of dyestuffs for their manufacturing purposes, and methylene blue (MB) is one of them. One of the most often utilized dyes in the manufacture of consumer goods, such as roasters, cutlery, and paper sheets, is MB (cationic azo dye). It may permanently harm the eyesight of people and animals alike, by causing severe eye burns. Acute palpitations and wheezing may be caused by some substances, which might exacerbate lung difficulties [6,7,8]. The investigation of suitable techniques for eliminating MB from wastewater discharge by the food sector is thus a fundamental challenge.

The adsorptive removal of dyes from industrial effluents, in particular, has been identified as one of the most workable and effective methods by virtue of its simplicity, high efficiency, and availability of pollutant capture sites in their structure [9,10,11]. Nowadays, electrospun-based nanofibers membrane materials, usually with smaller diameters (less than 100 nm) and larger surface areas, are extensively used to replace traditional adsorbents. The electrospinning process is carried out by applying an electric field (a high voltage power supply) in which the working solution contained in a syringe is connected to the spinneret using a needle, and the Taylor cone formed indicates the development of the nanofiber membranes. Subsequently, a stainless steel plate is used to collect the as-synthesized nanofiber membranes. To date, various polymers have been applied to fabricate nanofiber membranes because of their feasibility in the electrospinning process, meaning a greater adjustability of the diameter, alignment, and orientation in a linear form [12,13,14,15,16].

In comparison to other polymers, polyethersulfones (PESs) rely on their suitable thermal stability, mechanical strength, and chemical resistance, making them a suitable material in the realm of wastewater treatment. PESs have long been used to fabricate conventional commercial membranes because of their high permeability, affinity, and selectivity [17,18,19]. Accordingly, electrospun-based PES nanofiber membranes are currently applied at a large scale to remove pollutants from industrial effluents. For example, Koushkbaghi and co-workers [20] fabricated dual layers of chitosan/PVA/PES filled with aminated-Fe_3_O_4_ nanoparticles for the removal of Cr(VI) and Pb(II) ions. The adsorption capacity was strongly affected by solution pH. As such, pH 6 provides the maximum adsorption capacity for Cr(VI), while a lower pH was found to be suitable for maximum Pb(II) ions. In a similar approach, Zheng et al. [21] prepared ionic liquid grafted polyethersulfone-based electrospun nanofibrous membranes and demonstrated that developed membranes could be multifunctional materials, such as exhibited dye, heavy metals, and present antibacterial efficiency. Nevertheless, PES-based electrospun nanofiber membranes showed some limitations, such as hydrophobicity, low solubility, and stability, which need to be addressed in order to achieve a satisfactory performance.

Conversely, the usage of cellulose-based natural materials in electrospinning is on the rise because of their functionality, durability, and uniformity [22,23,24,25,26]. Among them, hydroxypropyl cellulose (HPC), a non-ionic ether of natural cellulose, is a polymer with temperature-dependent water solubility, excellent mechanical and thermal stability, and good chemical characteristics. It is becoming more popular because of its renewable ability, simplicity to manufacture, non-toxicity, and optical elements [27]. Additionally, surface wettability with an aqueous medium, heat resistance, and molecular transmission phenomena have been observed in HPC-based electrospun nanofiber membranes, making them desirable for wastewater treatment. For instance, Soraya Hassanpour et al. [28] reported that methylene blue (MB) dye was adsorbed from an aqueous solution using a new biocompatible adsorbent based on hydroxypropyl cellulose (HPC) and itaconic acid nanogels. For the phenol adsorption, composite hydrogels based on hydroxypropyl cellulose (HPC) and graphene oxide (GO) were developed and employed with a maximum adsorption capacity of 136.5 mg/g [29]. However, no one has yet reported on the synthesis of PES/HPC-based electrospun nanofiber membranes and their use in wastewater treatment; thus, further research is needed.

The present study, therefore, aims to fabricate a unique PES/HPC blended nanofiber membrane utilizing a one-step electrospinning scheme, and use it for the first time to remove the MB from an aqueous solution. Usually, the selected operational parameters, such as the initial solution pH, contact time, initial MB concentration, and ionic strength concentration, are carefully investigated in order to gain insights into the adsorption process.

## 2. Materials and Methods

### 2.1. Materials and Chemicals

In the present study, polyethersulfone (PES, Ultrason E 6020 P) with a molecular weight of 65,800 g/mol was purchased from BASF SE (Ludwigshafen, Germany). Hydroxypropyl cellulose (HPC, 99%, CAS No. 9004-64-2) was purchased from Shanghai Honest Chem. Co., Ltd. (Shanghai, China). *N*,*N*-dimethylacetamide (DMAc) (CAS No.: 127-19-5) was obtained from TNJ Chemical Industry Co., Ltd., (Hefei, China). Methylene blue (95% pure) was obtained from Sigma Aldrich Co., Ltd. (Darmstadt, Germany). A non-woven polyethylene terephthalate (PET) paper was acquired from Guocheng Co. (Wuxi, China) and used as a collector for the electrospun nanofiber membranes in this experiment. Other materials and reagents were employed without additional modification, and deionized (DI) water was used throughout the entire experiment.

### 2.2. One-Step Electrospinning

**Figure 1:**
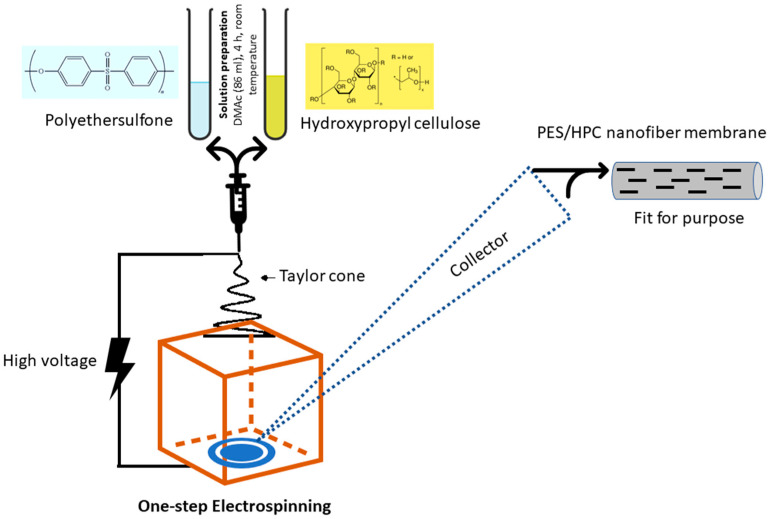
An illustration of the one-step electrospinning nanofiber membrane fabrication process.

As shown in Figure 1, the PES/HPC nanofiber membranes were produced using the one-step electrospinning process.

Blended PES/HPC solutions were prepared by dissolving 10 wt% PES and 2 different HPC concentrations (2 and 4 wt%) into 86 mL of DMAc solution, and continuously stirred for 4 h at room temperature. Then, the electrospinning process was carried out on a laboratory-size electrospinning machine (Foshan Lepton precision measurement and control technology Co., Ltd., M06, Foshan, China). The prepared solutions were transferred to separate 20 mL syringe pumps. Each solution was injected at a feeding rate of 1.0 mL/h under an applied voltage of 7.5 kV at 28 °C. The distance between the needle tip and the stainless steel plate was adjusted to 18 cm. The optimum electrospinning process conditions were determined by preliminary experiments. The nanofiber membranes were removed from the PET non-woven sheet and placed in a vacuum dryer oven (Zhengzhou Keda Machinery and Instrument Equipment Co., Ltd., Henan, China) for 5 h at 60 °C, to remove the DMAc solvent. The pristine PES nanofiber membranes were also made using the same procedure, for comparison purposes, but without the addition of HPC. Accordingly, the pristine HPC was also prepared using the absence of PES. Table 1 presents the overall electrospinning conditions.

### 2.3. Analytical Methods

An NDJ-8S digital rotating viscometer was used to measure the solution’s viscosity (Movel Scientific Instrument Co., Ltd., Ningbo, China). The pH was determined by a DZS-706A multi-parameter analyzer and the conductivity of the solution using a conductivity meter (INESA Scientific Instrument Co., Ltd., Shanghai, China). A professional DropMeter A-300 was used to measure the water contact angles of the membranes (Kudos Instruments Corp. New York, NY, USA). A Phenom desktop scanning electron microscope was used to analyze the nanofiber membrane surface morphologies (SEM, Thermo Fisher Scientific, Tokyo, Japan). The samples were gold-sputtered and the accelerating voltage was 5 kV prior to the acquisition of the SEM photos. The acquired SEM photos were utilized to analyze the fiber diameter distribution behavior of the membrane, which was processed using ImageJ software (https://imagej.nih.gov/ij/download.html, accessed on: 30 September 2021). Fourier transform infrared spectroscopy (IR, Interspectrum, low noise DLATGS, FTIR-920; Tartu maakond, Estonia) was used to detect the presence of functional groups in the nanofiber membranes. In order to obtain the quantitative FT-IR spectra, the samples were ground with potassium bromide powder in a mortar and pestle and then placed in front of the laser beam to be illuminated. Subsequently, the spectra were collected within the wavenumber ranging from 400 to 4000 cm^−1^. A TG analyzer (TG 209 F1 Libra, Netzsch Instruments, Wolverhampton, UK) was used to study the thermal characteristics of the samples throughout a temperature range of 0 to 850 °C. The heating rates were maintained at roughly 10°/min over the entire temperature range. The TG analysis was carried out at a flow rate of 50 mL/min, while the sample was in a nitrogen environment. The crystal structure of the membrane was determined using an X-ray diffractometer (Empyrean, Malvern PANalytical, Worcestershire, UK), with scans being taken from 2θ = 10° to 80°. The nanofiber membranes’ mechanical characteristics were assessed using an ASTM D882-18-compliant tensile tester (KD-III type BA-100m (Transcll technology, Shenzhen, China) at room temperature. The dimensions of the sample were 5 × 40 mm, and a rotational speed of 1 mm/min was employed throughout the test.

### 2.4. Batch Adsorption Studies

#### 2.4.1. The Effect of the Solution pH

The effect of the solution pH was investigated by the following experimental procedures: a total of 10 mg of weighted adsorbents was placed into VWR centrifuge tubes (polypropylene) holding 10 mL of a 400 mg/L MB solution and magnetically swirled for 24 h at a speed of 200 r/min under different pH conditions (3–10), at room temperature. The pH of the solution was adjusted by adding a buffer solution that had been previously made in the presence of 0.1 M HCl and NaOH. Following each test, a predetermined quantity of the solution was obtained at certain intervals and filtered through a 0.45 µm membrane filter. Following this, A Perkin-Elmer Lambda 25 UV-Vis spectrophotometer (Waltham, MA, USA) was used to measure the concentration of MB at a 200–800 nm wavelength range. The adsorption capacity of MB at time *t* (*q_t_*, mg/g) was calculated using the following Equation (1) [30]:

(1) $$q_{t}=\frac{{(C}_{0}-C_{t})V}{m}$$

where *C*_0_ (mg/L) and *C_t_* (mg/L) are the initial MB concentrations and that at time *t*, *V* (L) is the volume of MB solution, and *m* (g) is the adsorbent amount.

#### 2.4.2. The Effect of the Initial MB Concentrations

The effect of the initial concentrations of MB on the equilibrium adsorption capacity by the pristine PES and PES/HPC nanofiber membranes was examined. In order to create workable solutions for the different batch experiments, the stock solution was diluted in deionized water first. In order to assess the impact of the MB concentration on adsorption performance, a series of identical procedures were carried out under a range of different MB concentrations (200, 400, 600, 800, and 1000 mg/L) at a neutral pH and room temperature.

#### 2.4.3. The Effect of the Ionic Strength Concentration

In the presence of the produced pristine PES and PES/HPC nanofiber membranes, a standard adsorption approach was used to evaluate the influence of ionic strength on the adsorption capacity of MB. In a similar approach, 10 mg of adsorbents was put into 10 mL of 400 mg/L MB solution at a neutral pH by adding varying concentrations of NaCl (0.1–0.5 M) and stirred for 24 h.

### 2.5. Reusability

The reusability of the adsorbents was determined by soaking them in a 2 mM HCL solution for 6 h at room temperature. Five regeneration cycles were carried out under identical experimental conditions, with each tested adsorbent being washed twice with DI water and then prepared for the next adsorption cycle. Following that, the concentration of MB was measured to determine the adsorption capacity.

## 3. Results and Discussion

The current study was undertaken to develop a novel electrospun nanofiber membrane for the purification of food industry wastewater, whereby MB was used as a model pollutant. For this purpose, a non-toxic polymer hydroxypropyl cellulose was used as an additive to improve the affinity of the electrospun nanofiber membrane towards the purification medium, as indicated by the adsorption process. The structure–property relationship of the blended membrane was studied using a variety of characterization methods, and its adsorption behavior was investigated in depth, including the kinetics, isotherm, and reusability. Finally, a tentative adsorption mechanism was presented to explain the adsorption process.

### 3.1. The Properties of the Adsorbents

#### 3.1.1. SEM

**Figure 2:**
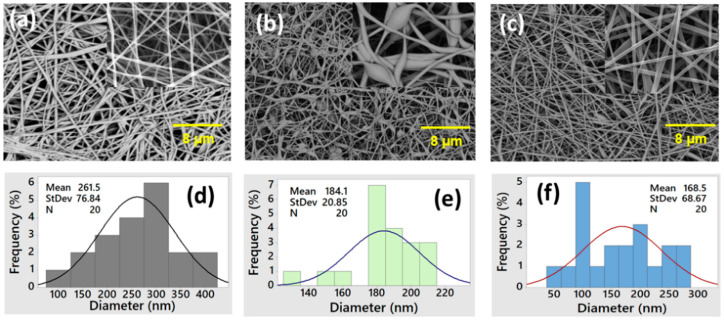
SEM images and average diameter distribution of the pristine PES (**a**,**d**), PES/HPC (10/2 wt%) (**b**,**e**), and PES/HPC (10/4 wt%) (**c**,**f**) nanofiber membranes.

SEM images were used to determine the analysis of the morphological traits of electrospun nanofiber membranes made of PES and PES/HPC, respectively. Regarding the average diameter distribution, the smooth surface of the pristine PES nanofiber membrane (Figure 2a) is characterized by homogeneous large-in-diameter fibers with a diameter of 261.5 nm (Figure 2d). On the other hand, blended PES/HPC nanofiber membranes (concentration: 10/2 wt%) (Figure 2b) produced smaller diameter fibers (184.1 nm) (Figure 2e), but beads were on their surface, which is not feasible. However, when the concentration of HPC was increased to 4% (Figure 2c), this resulted in a more homogeneous structure with a smaller diameter (168.5 nm) and more smooth fiber stacking (Figure 2f). In addition, the reason for this phenomenon might be described by decreasing the solution viscosity (Table 1), which, in turn, reduces the nanofiber diameter; increasing the solution conductivity (Table 1) also has the same effect of reducing the fiber diameter, as reported elsewhere [31,32]. Therefore, in view of the smaller diameter and beads-free membrane, pristine PES and PES/HPC (10/4 wt% concentration) samples were chosen for the following experimental parts. Moreover, it is known that composite polymer nanofiber membranes with smaller diameters have a high specific surface area at a given volume than those with a larger diameter, and hence more active sites to adsorb more organic pollutants during adsorption [33,34].

#### 3.1.2. FTIR

**Figure 3:**
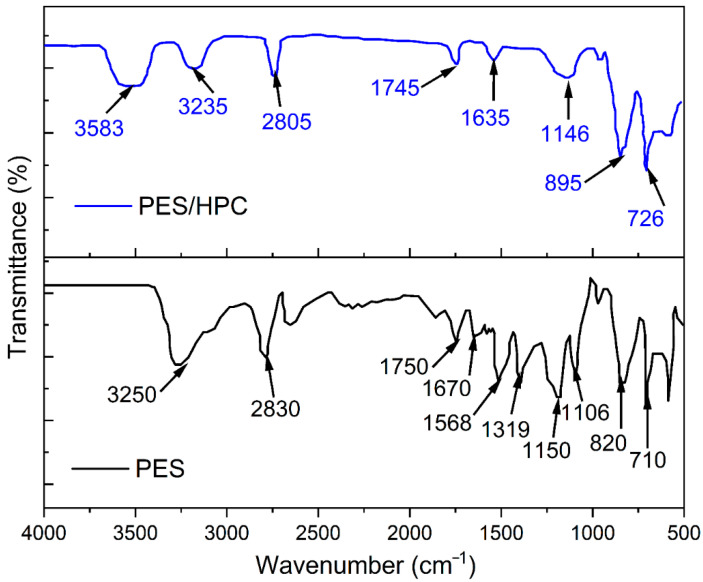
FTIR spectra of the PES and PES/HPC nanofiber membranes.

FTIR is well known for its effectiveness in interpreting structural data by providing the vibrational band’s shape, intensity, and changes of environmental and conformation characteristics at the molecular level of polymers. Figure 3 depicts the FTIR spectra of the pristine PES and the blended PES/HPC electrospun nanofiber membrane. From the pristine PES nanofiber membrane spectrum, it was observed that the absorption peaks appeared at 710 cm^−1^ and 820 cm^−1^, assigned to CH_2_ bond C–H stretching, respectively. The characteristic bands of the functional groups at 1106–1150 cm^−1^, 1319 cm^−1^, 1568 cm^−1^, 1670 cm^−1^, and 1750 cm^−1^ correspond to O=S=O, C–O stretching, C=C stretching, N–C=O carbonyl vibrations, and C=O stretching, respectively [35]. The stretching vibration of aromatic C–H groups is also responsible for the two prominent absorptions broader peaks observed at 2830 cm^−1^ and 3250 cm^−1^ [36]. The absorption peaks of the blended PES/HPC membrane are almost identical to those of the pristine PES nanofiber membrane, except for smaller additional peaks across the blended membrane spectrum. For example, the wide absorption peak at 3583 cm^−1^ is attributed to the OH stretching vibration of free hydroxyl and hydrogen bonds. Subsequently, a shoulder peak at 1635 cm^−1^ (C=O stretching), 1164 cm^−1^ (C–O asymmetric stretching), and two consecutive sharp narrow peaks were counted at 895 cm^−1^ (C–O deformation and -CH_2_ rocking) and 726 cm^−1^ for the twisting of O–H, which are typical of the nature of HPC polymers [37,38]. Based on these findings, it can be concluded that the successful interaction between PES and HPC was dominated by physical contact rather than a chemical reaction.

#### 3.1.3. XRD

**Figure 4:**
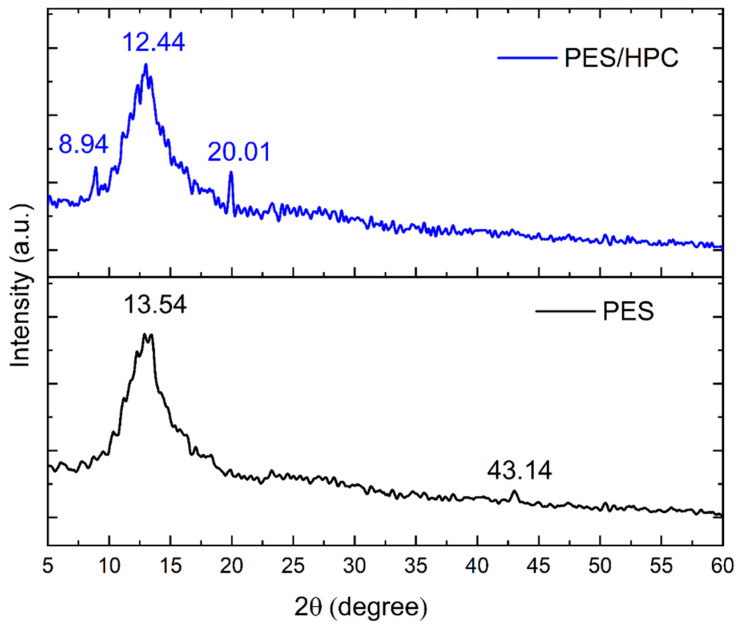
The XRD pattern of the PES and PES/HPC nanofiber membranes.

Figure 4 shows the XRD patterns for the pristine PES and PES/HPC blended electrospun nanofiber membrane. Due to the amorphous nature of the PES polymer, the pure PES nanofiber membrane displayed a strong characteristic peak at 2θ = 13.54°. Additionally, there was another peak that emerged at 2θ = 43.14° because of the second carbon pair existence in the neighboring chain [39]. The effect of the addition of HPC on the blended PES/HPC nanofiber membrane did not present any major changes, with a broader peak at 2θ = 12.44°. It is considered that the somewhat ordered amorphous phase of the HPC is responsible for the wide peak at 2θ = 20.1°, while the crystalline phase of the main chain backbone–backbone d-spacing corresponds to the peak at 2θ= 8.94° [40].

#### 3.1.4. TGA

**Figure 5:**
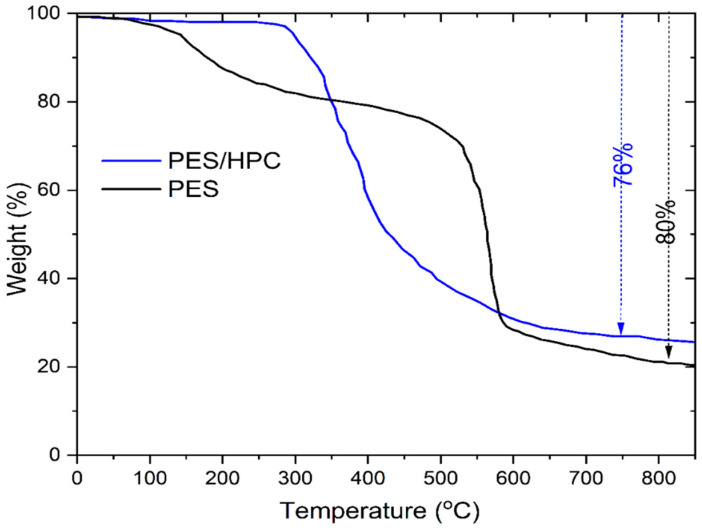
The TGA curves of the PES and PES/HPC nanofiber membranes.

Figure 5 displays the results of a TGA profile on the thermal stability of the pristine PES and the blended PES/HPC electrospun nanofiber membranes, and is nowadays receiving special attention. The pristine PES nanofiber membrane exhibited three phases of weight loss, whereby the weight loss in the first phase (30–100 °C) was less than 5% because of the evaporation of water molecules that were physically attached to the polymer backbone chain. Following this, the elimination of the leftover solvent from the pristine nanofiber membrane surface occurred during the second-phase degradation, which took place between 140 and 400 °C, and resulted in a weight loss of 21%. Additionally, when deterioration reached a temperature between 496 and 673 °C, the ether bond (C–O) fracture of the pristine PES nanofiber membrane caused the greatest weight loss (around 54%) [41]. The case of the PES/HPC blended electrospun nanofiber membrane is interesting; it exhibits a two-phase thermal degradation pattern, and a modest weight loss between (1–5)% was detected during the first phase (30–100 °C) due to moisture evaporation. Additionally, the major weight loss occurred from 285 to 650 °C (around 71%), with a maximum degradation peak found at 355 °C, which is related to the existence of the DMAc solvent evaporation temperature. Moreover, this degradation pattern was much lower than the HPC polymer reported in the literature [42]. Overall, it was observed that the blended PES/HPC nanofiber membrane demonstrated a better thermal performance (total weight loss: 76%), compared to the pristine PES nanofiber membrane (total weight loss: 80%). These findings indicate the existence of a physical interaction between hydroxypropyl cellulose and PES materials. Furthermore, more hydrogen bonds were established between the hydroxyl groups of HPC and the sulfone groups of PES in the presence of HPC as an adhesion material, which increased PES/HPC interfacial contact and promoted thermal stability [43]. This observation is consistent with an earlier study that found blended nanofiber membranes acceptable for thermally coupled wastewater-treatment applications [44].

#### 3.1.5. Mechanical Properties

**Figure 6:**
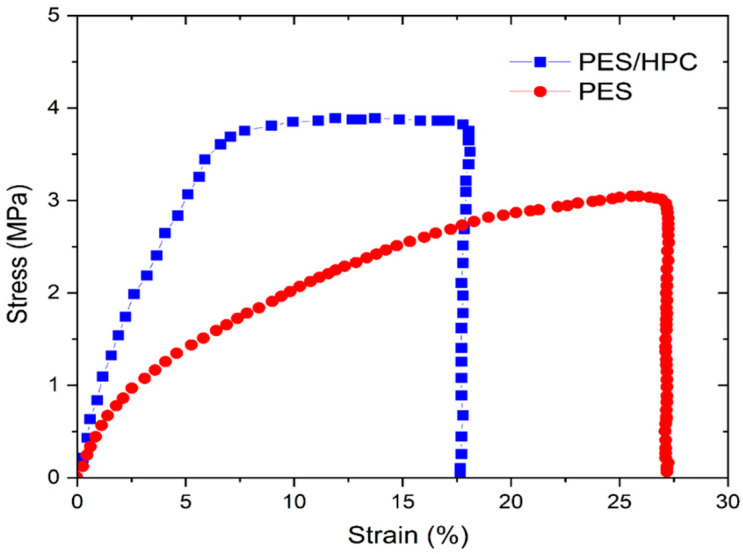
The stress–strain curve of the PES and PES/HPC nanofiber membranes.

The mechanical properties of the electrospun NMs have been proven to be critical in wastewater treatment [45]. The mechanical characteristics between pristine PES and blended PES/HPC nanofiber membranes were examined in this study using tensile studies, and the findings are presented in Figure 6. A larger strain percentage (27.12%) was observed in the pristine PES nanofiber membrane, although the tensile strength of the membrane was more reduced (around 3 MPa). A 30.2% increase in the tensile strength (3.82 MPa) was observed in the blended PES/HPC nanofiber membrane, compared to the pristine PES nanofiber membrane, demonstrating that the intercalation of HPC into the PES matrix significantly improves the intermolecular interaction. Additionally, because of the presence of surface hydroxyls, it is possible to increase the number of physical or chemical cross-linking sites, which is favorable to the mechanical qualities [46]. Additionally, a larger number of nodes may be found in the fiber pores, thanks to a reduction in the overall diameter of the blended PES/HPC nanofiber membrane due to HPC inclusion (an increased surface area), resulting in higher tensile strength.

#### 3.1.6. Hydrophilicity

**Figure 7:**
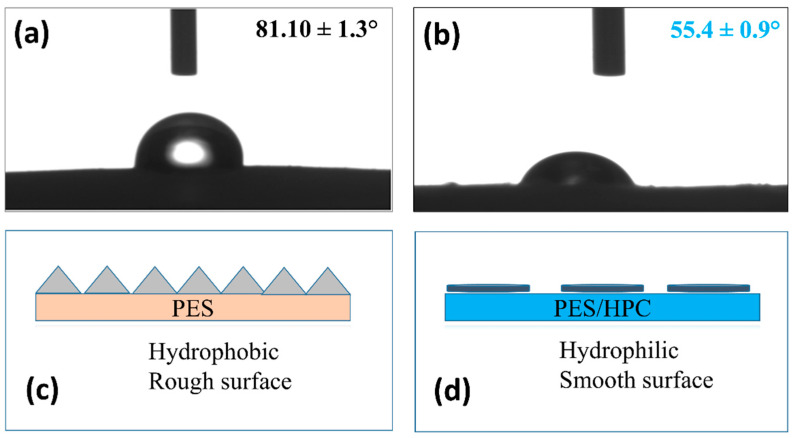
The water contact angle of the pristine PES (**a**,**b**) PES/HPC nanofiber membranes, with their surface states (**c**,**d**), respectively.

The membrane’s surface wettability, particularly its hydrophilicity, is thought to be a significant characteristic for improving water purification performance [47]. This study assessed the hydrophilicity of two electrospun nanofiber membranes by measuring their contact angles. For the pristine PES electrospun nanofiber membrane, the contact angle was 81.10 ± 1.3° (Figure 7a), which indicates that the membrane had less hydrophilicity with a rough surface (Figure 7c). A lower contact angle of 55.4 ± 0.9° was found for the blended PES/HPC electrospun nanofiber membrane, indicating that the membrane has a more hydrophilic and smooth surface (Figure 7b,d). The contact angle of the membranes tends to decrease when a HPC polymer is added to the solution, as reported in a previous study by Gradinaru et al. [48]. Overall, this study implies that, because of their hydrophilicity, PES/HPC membranes adsorb more dye molecules, utilizing their high porosity behavior to generate a stronger affinity between the water/dye and membrane surface [29,49].

### 3.2. MB Adsorption Studies

#### 3.2.1. The Effect of the Initial Concentration

**Figure 8:**
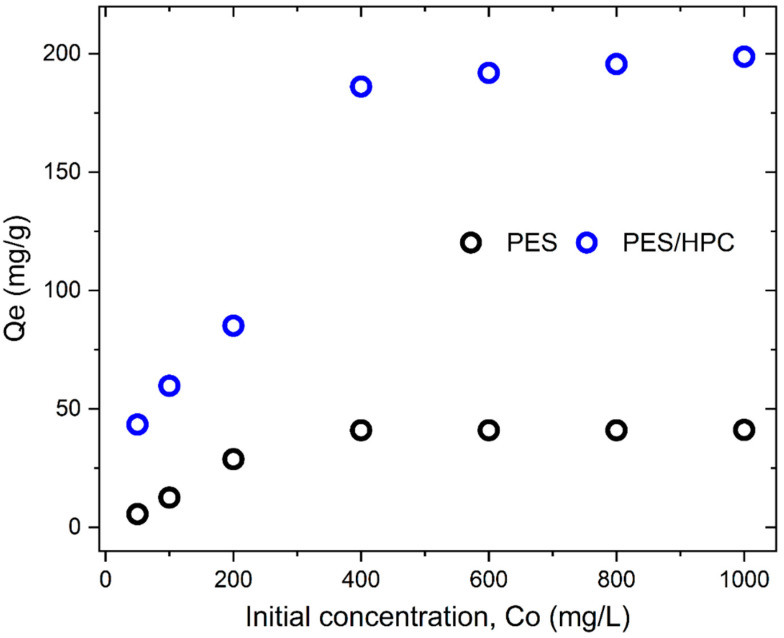
The effect of the initial concentrations on the MB adsorption capacity of the PES and PES/HPC nanofiber membranes.

The adsorbent’s binding sites are influenced by the initial dye concentration, which has an indirect effect on dye adsorption capability. Consequently, the adsorption capacity was tested under optimum conditions at various starting concentrations ranging from 50–1000 mg/L. The adsorption capacity of both adsorbents is favorably influenced by the starting concentration, as is shown in Figure 8.

The fast adsorption capacity (43.47–186.12 mg/g) of a PES/HPC blended nanofiber membrane was observed with an increase in the starting concentration from 50–400 mg/L, while increasing the concentration further, by up to 1000 mg/L, resulted in a slightly increased adsorption capacity of 198.78 mg/g. When the concentration is more than 400 mg/L in both scenarios, a reasonable increase in the adsorption capacity indicates that equilibrium has been attained. A similar pattern was observed for the pristine PES nanofiber membrane, although the adsorption capacity was relatively low throughout the entire varied concentrations, where the highest capacity of 41.02 mg/g was achieved at 400 mg/L. This result indicates that an increase in the concentration promotes mass transfer, which, in turn, raises the driving force for MB adsorption. MB molecules are also transported in significant numbers from the aqueous phase to the nanofiber membrane’s solid surface, resulting in an increased adsorption capacity [50].

#### 3.2.2. Adsorption Kinetics

**Figure 9:**
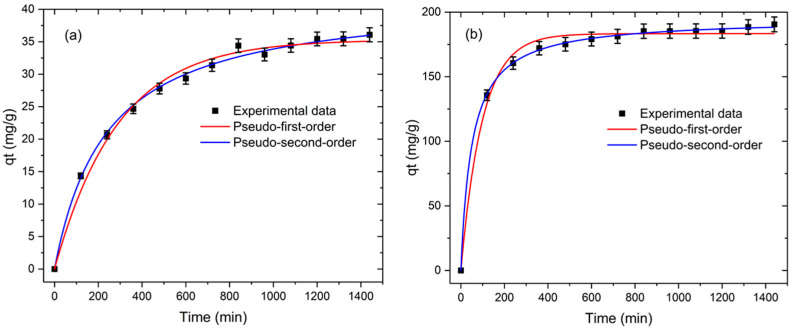
The pseudo-first-order and pseudo-second-order kinetic models for the MB adsorption on PES (**a**) and PES/HPC (**b**).

The study of adsorption kinetics is crucial for determining the rate constant of the entire adsorption process in relation to the contact time. Therefore, the kinetic behavior of the MB adsorption onto PES and PES/HPC membranes was studied, with the findings presented in Figure 9a,b, respectively. At the start of the experiment, the adsorption rate was rapid, but it steadily slowed down as the duration continued, until it reached equilibrium. The equilibrium for the MB adsorption was attained in 1080 min; it took 1440 min with PES, but only 840 min with the PES/HPC, whereby 75% of MB was adsorbed within 360 min. These findings show that the introduction of HPC into PES resulted in a surface area with more MB-capture active sites.

Moreover, nonlinear pseudo-first-order (PFO) (Equation (2)) and pseudo-second-order (PSO) (Equation (3)) models were used to explain the adsorption kinetics.

(2) $$q_{t}=q_{e}\left(1-e^{-k_{1}t}\right)$$

(3) $$q_{t}=\frac{k_{2}q_{e}^{2}t}{1+k_{2}q_{e}t}$$

where *q_t_* (mg/g) represents the adsorption capacity of MB at any time *t*; *k*_1_ (min^−1^) and *k*_2_ (g/mg/min) are the kinetic rate constants for the pseudo-first order and pseudo-second order, respectively.

Figure 9a,b illustrates the fitted curves, with the fitted values reported in Table 2. For PES, PSO had a higher correlation co-efficiency PSO (R^2^ = 0.9965) than PFO (R^2^ = 0.9893). When the PES/HPC blended nanofiber membrane was present, the correlation co-efficiency of PFO (R^2^ = 0.9908) was lower than that of PSO (R^2^ = 0.9995) in terms of R^2^. MB adsorption onto the PES and PES/HPC nanofiber membranes was found to be well fitted by the PSO kinetic model, suggesting that the adsorption process of MB is dominated by the chemisorption mechanism, in which electron exchange occurs between the adsorbent and MB molecule binding sites, rather than by the electrostatic mechanism. Previous investigations have also shown that the pseudo-second-order kinetic model for MB adsorption may have a good fitting. For example, Luo et al. [51] observed that the adsorption of MB followed a second-order kinetic model when they used a cellulose nanofiber-based highly flexible compressible super assembled aerogel. Based on our research, it can be concluded that the PES/HPC nanofiber membrane demonstrates an outstanding molecular adsorption performance of MB in a short period of time, making it suitable for practical use.

#### 3.2.3. Adsorption Isotherms

Adsorption isotherms are a key factor in the adsorption process. Using this scheme, the maximum adsorption capacity can be identified by determining the relationship between the adsorbent and adsorbate equilibrium concentrations. Herein, the adsorption behavior is investigated using two widely used nonlinear isotherm models, namely the Langmuir model (Equation (4)) and the Freundlich model (Equation (5)). The Langmuir fitted model indicates that the adsorption process occurs monolayer onto the homogeneous solid adsorbent surface, while the Freundlich fitted isotherm model indicates that the adsorption process occurs multilayer onto the heterogeneous solid adsorbent surface [52,53].

(4) $$q_{e}=\frac{q_{\max}k_{L}C_{e}}{1+k_{L}C_{e}}$$

*q_e_* = *K_F_C_e_*^1/^*^n^* (5)

where *q_max_* denotes the maximum adsorption capacity of MB (mg/g); *K_L_* represents the constant of the Langmuir equation; *Ce* is used for the measurement of the solution concentration at equilibrium; 1/*n* highlights the intensity of the adsorption; and *K_F_* indicates the constant of the Freundlich equation.

**Figure 10:**
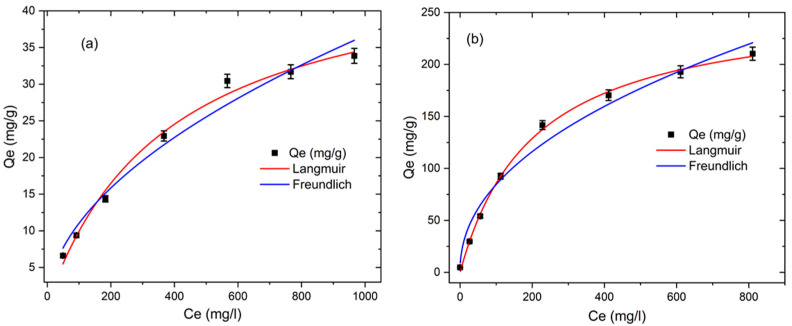
The adsorption isotherms of MB by PES (**a**) and PES/HPC (**b**), according to the Langmuir and Freundlich equations.

A set of fitted curves are shown in Figure 10, while a list of derived isotherm parameters can be found in Table 3. Based on the correlation co-efficiency values (R^2^), the adsorption isotherms for both membranes were consistent with the Langmuir isotherm model, demonstrating monolayer adsorption on the heterogeneous surface of adsorbents [9]. According to the results, the PES/HPC nanofiber membrane has a maximum MB adsorption capacity of 259.74 mg/g, while the pristine PES nanofiber membrane has a capacity of just 48.00 mg/g. This implies that the PES/HPC nanofiber membrane considerably increased the adsorption capacity of MB, which is integrated with the *K_L_* value of the adsorbent, since a higher *K_L_* of the adsorbent results in an improved adsorption performance at a low concentration [54]. Table 4 compares our adsorbent to other adsorbents for MB adsorption and shows that PES/HPC has an excellent adsorption capacity, and it is much higher than others. The exceptional performance of the PES/HPC nanofiber membrane might be explained by the presence of sulfonic and hydroxyl groups on the membrane’s surface, which electrostatically interact with the cationic site of MB. This shows that the PES/HPC nanofiber membrane may be a highly efficient adsorbent for removing pollutants from wastewater.

#### 3.2.4. Adsorption Mechanism

**Figure 11:**
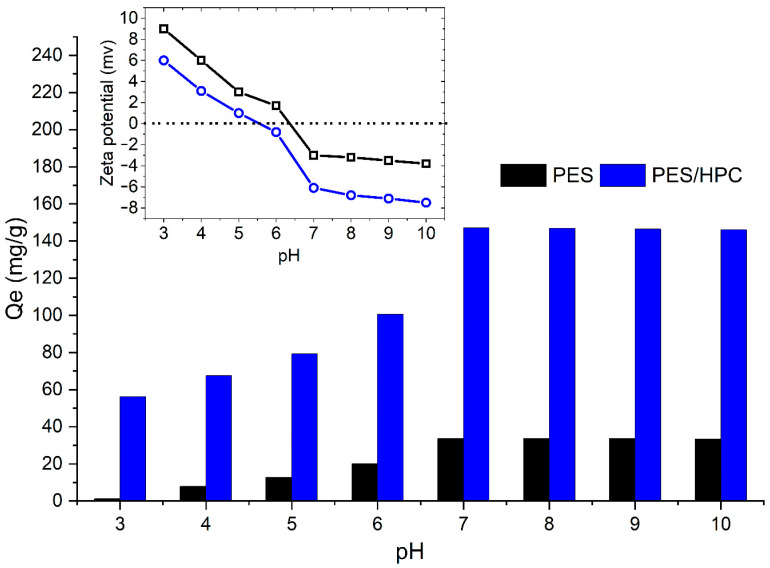
The effect of the pH on the MB adsorption capacity.

The pH of the solution has been thought to be important, because the surface charge of adsorbents and the adsorbate are very dependent on the pH values determining the adsorption performance. Consequently, the MB adsorption capacity was examined in the presence of the PES and PES/HPC nanofiber membranes at different pH values ranging from 3 to 10, and the findings are presented in Figure 11. With respect to both membranes, the pH range between 3 and 5 was found to be steady, while the pH value rose from 5 to 7 and then progressively attained the greatest MB adsorption capacity between 7 and 10 for both membranes. In particular, the highest adsorption capacity of MB was 33.68 mg/g and 147.09 mg/g for PES and PES/HPC, respectively, at a neutral pH, which is in line with previously published research [62]. The reason for these results can be explained as follows: (1) the protonation behavior of the existing functional groups causes the nanofiber membrane surface to become positively charged at an acidic pH. As a result of the electrostatic repulsion between the positively charged membrane surface and the cationic MB molecules, the adsorption uptake was decreased. (2) On the contrary, when the pH is changed to neutral or alkaline, the nanofiber membrane surface charge exhibits greater negativity, leading to a higher adsorption capacity. In the case of the blended PES/HPC membrane, sulfonic and hydroxyl groups deprotonate at a neutral pH, resulting in the membrane’s negative charge [43]. Overall, it was shown that at higher pH levels, the PES/HPC membrane developed an electrostatic attraction ionic interaction with the positive-charged MB molecules, which resulted in a tendency towards a greater adsorption capacity, especially at neutral pH levels. Furthermore, the zeta potential, as shown in Figure 11 (inset), provides additional evidence of these occurrences. For the PES and PES/HPC nanofiber membranes, the point of zero charge (pzc) was observed at pH 6.3 and 5.5, respectively. Thus, both membranes have a positive <pzc and negative >pzc surface charge, with the negatively charged surface favoring adsorption through electrostatic attraction, as is evident in our experimental study.

**Figure 12:**
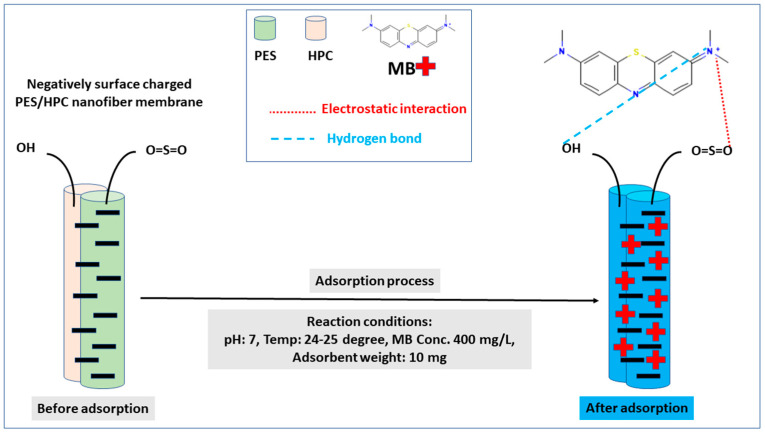
The proposed adsorption mechanism of MB in the presence of the PES/HPC nanofiber membrane.

Based on the above discussion, the tentative adsorption mechanism is depicted in Figure 12. In adsorption, dye is adsorbed by the nanofiber membrane, and it is essential to establish the mechanism that provides this desired adsorption capacity. Sulfonyl functional groups are enriched in the backbone of the polyethersulfone compound and interact with a cationic MB dye through various forces, such as an electrostatic interaction, hydrogen bond, and Π–Π interaction [63]. The presence of sulfone groups, which are more polar than ether groups, results in a more charged exchange between the adsorbent and adsorbate, thus enhancing the electrostatic attraction and leading to a noticeable improvement of the adsorption capacity for the PES/HPC nanofiber membrane [64]. It is worth mentioning that the synthesized PES/HPC nanofiber membrane demonstrated an improved adsorption capability at a neutral pH, which implies that the blended membrane surface includes a significant number of oxygen-containing functional groups, which are deprotonated at higher pH levels and facilitate electrostatic adsorption. The addition of HPC also provided hydroxyl groups and facilitated the adsorption process by making both an electrostatic interaction and hydrogen bonding and contributed to the adsorption process. Furthermore, the presence of ionic strength decreased the adsorption capacity (Table 5), which is another form of proof that the electrostatic interaction was dominant for MB adsorption by the blended PES/HPC nanofiber membrane.

### 3.3. The Effect of Ionic Strength

A wide range of contaminants, such as suspended and dissolved chemicals, acids or alkalis, salts, metal ions, and other hazardous substances, may be found in food manufacturing wastewater. The presence of ions raises the ionic strength of the solution, which might affect the efficacy of the adsorption process. Thus, NaCl was added to the solution in variable concentrations to study the influence of ionic strength on the adsorption capacity of MB in the PES and PES/HPC nanofiber membranes (Table 5). It was noticed that when the concentration of NaCl was increased, the MB adsorption capacity was reduced simultaneously. This could be attributed to the evolution of the electrostatic repulsion between the dye molecules and negatively charged adsorbent surfaces at higher NaCl concentrations; as the Na^+^ ions of NaCl compete for binding sites on the membrane surface with cationic MB, less adsorption occurs. A similar phenomenon was also observed for MB adsorption on a cellulose-based bio adsorbent, as reported by Liu et al. [65].

### 3.4. Reusability

Commercially accessible adsorbers can only compete if they can be used repeatedly and can be regenerated. Adsorbents are judged to be effective, based on the presence of these characteristics. As a result, a series of experiments were carried out employing PES and PES/HPC membranes for up to five cycles under optimum conditions (Table 5). According to the results, the pristine PES nanofiber membrane lost 32.21% of its adsorption capacity after 5 cycles, but the PES/HPC nanofiber membrane lost only 4.51%, indicating that the blended PES/HPC membrane is more reusable. For the PES/HPC nanofiber membrane, the smaller fiber diameter (resulting in a greater surface area) and increased hydrophilicity are credited with its exceptional performance. Given its high recyclability, the PES/HPC nanofiber membrane adds another important property to its wastewater treatment adsorption capacity.

## 4. Conclusions

As an additive, it is claimed that HPC has a potential use in treating industrial wastewater. HPC was successfully used to develop a new PES-based electrospun nanofiber membrane in the current study. According to the morphological evidence, the synthesized PES/HPC blended nanofiber membrane was devoid of beads and had an ultrathin diameter (168.5 nm), in comparison to the original PES nanofiber membrane (261.5 nm). Furthermore, it displayed increased hydrophilicity, thermal stability, and mechanical stability, all of which are important properties in the adsorption process. Additionally, it demonstrated increased hydrophilicity as well as thermal and mechanical stability, all of which are critical in the adsorption process. The PES/HPC nanofiber membrane took only 840 min (where 75% of the MB was adsorbed within 360 min) to achieve equilibrium, while 1080 min was required for the pure PES nanofiber membrane, and the kinetics data revealed that the pseudo-second-order (PSO) model performed much better than the pseudo-first-order (PFO) model. The findings of the adsorption isotherms revealed that the PES/HPC blended nanofiber membrane has an excellent MB adsorption capacity of 259.74 mg/g at a neutral pH at room temperature, which is more superior, in comparison to the pure PES nanofiber membrane (48.00 mg/g) obeying the Langmuir model. With regard to the ionic strength, the adsorption capacity of the PES/HPC decreased with increased ionic strength concentrations, but the recyclability was maintained for up to 5 cycles with good adsorption results. Overall, it can be concluded that the newly synthesized PES/HPC electrospun nanofiber membrane proves to be a very effective adsorbent for absorbing organic pollutants, such as MB, and can also be used for capturing other pollutants, such as bacteria, phosphorus, ammonia, nitrogen, and viruses from industrial wastewater effluents, which is of paramount importance for clean water production and environmental sustainability.

## Figures and Tables

**Table 1:** The electrospun solution properties and their fiber characteristics.

Sample Type	Polymer Concentrations (wt%)	Voltage (kV)	Flow Rate (mL/h)	Viscosity (mPa/s)	Conductivity (mS/cm)	Fiber Morphology	Diameter (nm)
PES	10	7.5	1.0	2268	1.6	Continuous fibers	261.5
HPC	2	12	1.5	320	0.002	No fibers	-
PES/HPC	10/2	7.5	1.0	1845	1.9	Beads with fibers	184.1
PES/HPC	10/4	7.5	1.0	1543	2.2	Continuous fibers	168.5

**Table 2:** The kinetic parameters for MB adsorption.

Samples	Pseudo-First Order			Pseudo-Second Order		
	K_1_ (min^−1^)	*q_e_* (mg/g)	R^2^	K_2_ (g/mg/min)	*q_e_* (mg/g)	R^2^
PES	0.0034	35.3161	0.9893	0.0001	41.9531	0.9960
PES/HPC	0.0101	183.3293	0.9908	0.0004	195.0212	0.9995

**Table 3:** The adsorption isotherm parameters for MB.

Samples	Langmuir			Freundlich		
	$q_{max}$ (mg/g)	*K_L_* (L/mg)	R^2^	*K_F_* (mg/g)	1/*n*	R^2^
PES	48.0076	0.0026	0.9913	1.0012	0.5211	0.9747
PES/HPC	259.7402	0.0049	0.9984	10.1847	0.4593	0.9693

**Table 4:** A comparison of the MB adsorption capacity with the previously reported literature.

Adsorbent	Optimum MB Conc. (mg/L)	Optimum pH	Kinetics	Isotherm	*q_max_* (mg/g)	Ref.
Cellulose nanofibrils	100	9	-	Langmuir	122	[55]
Deacetylated cellulose acetate (DA)@polydopamine (PDA) nanofibers	50	6.5	2nd order	Langmuir	88.2	[56]
Graphene/TEMPO-oxidized cellulose nanofibrous	100	6.5	2nd order	Langmuir	227.27	[57]
Cellulose citrate	100	3	2nd order	Langmuir	96.2	[58]
Hydroxypropyl cellulose (HPC)/graphene oxide hydrogels	-	-	2nd order	Freundlich	118.4	[59]
Cellulose sponge	30	7	2nd order	Langmuir	123.46	[60]
Vanadium pentoxide (V_2_O_5_) nanoparticles/PES	1	10	2nd order	Freundlich	85%	[61]
PES nanofibers	400	7	2nd order	Langmuir	48.0	Present work
PES/HPC nanofibers	400	7	2nd order	Langmuir	259.74	Present work

**Table 5:** The effect of NaCl concentrations and reusability tests on the MB adsorption capacity by the PES and PES/HPC nanofiber membranes, respectively.

Samples	Control, *q_e_* (mg/g)	NaCl Concentrations (M), *q_e_* (mg/g)				
		0.1	0.2	0.3	0.4	0.5
PES	32.47	29.44	27.34	23.77	21.88	20.44
PES/HPC	185.45	182.32	180.21	179.15	178.05	177.25
		Cycles, *q_e_* (mg/g)				
		1	2	3	4	5
PES	32.47	30.17	27.14	24.05	22.13	20.45
PES/HPC	185.45	183.12	181.88	178.18	176.21	174.85

## Data Availability

The datasets generated during the current study are available from the corresponding author on reasonable request (Vincenzo Naddeo, V.N.).
